# Gas-sensing features of nanostructured tellurium thin films

**DOI:** 10.3762/bjnano.11.85

**Published:** 2020-07-10

**Authors:** Dumitru Tsiulyanu

**Affiliations:** 1 CIMAN Research Centre of Department of Physics, Technical University, bul. Dacia 41, MD-2060 Chisinau, Moldova

**Keywords:** gas-sensing properties, NO_2_, tellurium thin films, nanocrystalline films

## Abstract

Nanocrystalline and amorphous nanostructured tellurium (Te) thin films were grown and their gas-sensing properties were investigated at different operating temperatures with respect to scanning electron microscopy and X-ray diffraction analyses. It was shown that both types of films interacted with nitrogen dioxide, which resulted in a decrease of electrical conductivity. The gas sensitivity, as well as the response and recovery times, differed between these two nanostructured films. It is worth mentioning that these properties also depend on the operating temperature and the applied gas concentration on the films. An increase in the operating temperature decreased not only the response and recovery times but also the gas sensitivity of the nanocrystalline films. This shortcoming could be solved by using the amorphous nanostructured Te films which, even at 22 °C, exhibited higher gas sensitivity and shorter response and recovery times by more than one order of magnitude in comparison to the nanocrystalline Te films. These results were interpreted in terms of an increase in disorder (amorphization), leading to an increase in the surface chemical activity of chalcogenides, as well as an increase in the active surface area due to substrate porosity.

## Introduction

Tellurium (Te) is a multifunctional chemical element used for the development of many devices, such as diodes with high (10^6^) rectification ratios, thin-film field-effect transistors, optical recording media, infrared and UV detectors, strain-sensitive devices and others (see [1–2] for extended reviews on the topic). In the last decade, Te has also become an attractive element with great biological applicability since it can be used as quantum dots in imaging and diagnostics and has antibiotic properties [3]. Even though Te has a biological relevance, it is largely used in the development of thin films in chemical-sensing applications, especially for toxic gas sensing. Szaro [4] pioneered the studies regarding the effects of oxygen and nitrogen, diluted in either dry or wet air, on the electrical properties of Te films. The results showed an increase in the hole concentration during the adsorption process but not in the mobility of these holes. However, the changes in the electrical properties induced by these gases were very small and irreversible and were, later on, confirmed and explained in a systematic, relevant work [5]. In the early 2000s, Tsiulyanu and coworkers [6] started to thoroughly investigate the use of Te thin films as an active element in gas sensor manufacturing. They showed that microcrystalline Te thin films, grown by thermal vacuum evaporation, exhibit high sensitivity to low concentrations (ppm range) of nitrogen dioxide (NO_2_) even at room temperature. Subsequent studies showed that it was possible to increase the concentration range sensitivity to more than 300 ppm NO_2_ by growing single-crystalline microtubes. In order to do that, Te metal was evaporated onto quartz substrates under an inert argon gas at ambient pressure [7]. Later, it was also found that microcrystalline Te films have remarkable, sensitive properties toward ammonia [8–9] and hydrogen sulfide [10] and, to a lesser extent, to carbon oxides and amines [11]. In the last years, due to the increase in the general interest toward nanodimensional devices and structures, significant attention has been given to growing, studying and applying nanostructured Te. To achieve these goals, different and sometimes quite sophisticated chemical, electrochemical and physical methods have been developed. In line with this, Wang and collaborators [12] used thermal decomposition of Te dietyldithiocarbamato film to grow Te nanoflakes. In order to synthesize Te nanowires, Liang and collaborators [13] performed chemical reactions of Na_2_TeO_3_, in aqueous solution, via hydrothermal treatment, whereas Ma and colleagues [14] used a solvothermal approach on glass substrates. To synthesize Te nanotubes, techniques such as galvanic displacement of sacrificial cobalt nanowires were employed [15]. Lastly, to grow one-dimensional nanostructures, either template-free electrodeposition of Te, from an ionic liquid binary mixture [16], or thermal evaporation in a furnace under argon gas flow [17] were strategies utilized.

The present work is related to investigations of the interaction between nanostructured Te films and toxic gases. According to the literature, such investigations firstly have been provided utilizing the nanocrystalline Te films grown onto Pyrex glass, alumina (Al_2_O_3_), oxidized silicon or sapphire substrates via thermal vacuum evaporation of pure Te [18–20]. Tests were performed in those films in order to access their ability to detect both oxidizing (NO_2_) and reducing (H_2_S) toxic gases at temperatures between 77 and 423 K. Depending on the experimental conditions in which the films were prepared (such as temperature, type and concentration of the target gas), their sensing characteristics were found to vary. For instance, the best response and recovery time values toward NO_2_ were around 30 s and 7 min, respectively, at 40% sensitivity (defined as the relative variation of the resistance). Such sensing parameters did not differ much from the similar parameters obtained earlier for microcrystalline Te films. Further investigations have been extended to Te nanotubes grown on quartz or Si(111) substrates through a catalyst-free growing process in a furnace filled with argon [21]. Another study used the high-vacuum deposition technique in order to grow Te nanotubes on silicon substrates containing previously deposited nanoparticles of silver or gold [22]. In both cases, 50 nm diameter Te nanotubes were obtained. When exposed to low concentrations of different toxic gases, including NO_2_, the Te nanotube-based sensors showed similar (or sometimes lower) numbers regarding sensitivity and response/recovery times in comparison to Te single-crystalline microtube-based gas sensors [7]. An increase in the gas-sensing performance was achieved by growing single-crystal Te-based nanotubes and nanowires via hydrothermal recrystallization [23]. The response time range of NH_3_ gas sensors based on such nanocomponents was 5–18 s but the recovery time ranged between 170–720 s. From comparison with state-of-the-art devices, it can be observed that the physically nanostructured Te thin films exhibit great potential for applications in development in advanced gas sensors and, so far, are the only Te-based nanostructured sensors tested with this purpose. Besides, it can also be observed that nanostructuring is mostly performed via phase transformations, such as hydrothermal recrystallization and growth of Te nanocrystals, nanotubes or nanowires from the gas phase under vacuum or argon atmosphere. On the other hand, nanostructuring can be performed mechanically as indicated by the possibility of growth of nanocrystalline gas sensors via rf sputtering (13.6 MHz) of Te in an ultra-high-purity argon atmosphere [24].

The main aims of the present work were to investigate and improve the gas-sensing parameters of nanostructured Te films by using a mechanical nanostructuring approach. Crystalline and amorphous Te films were grown, respectively, on glass or porous, nanostructured, dielectric substrates. These two physically nanostructured Te films were studied with a special focus on the gas-response kinetics.

## Results and Discussion

### Sample preparation, morphology and structure

**Figure 1:**
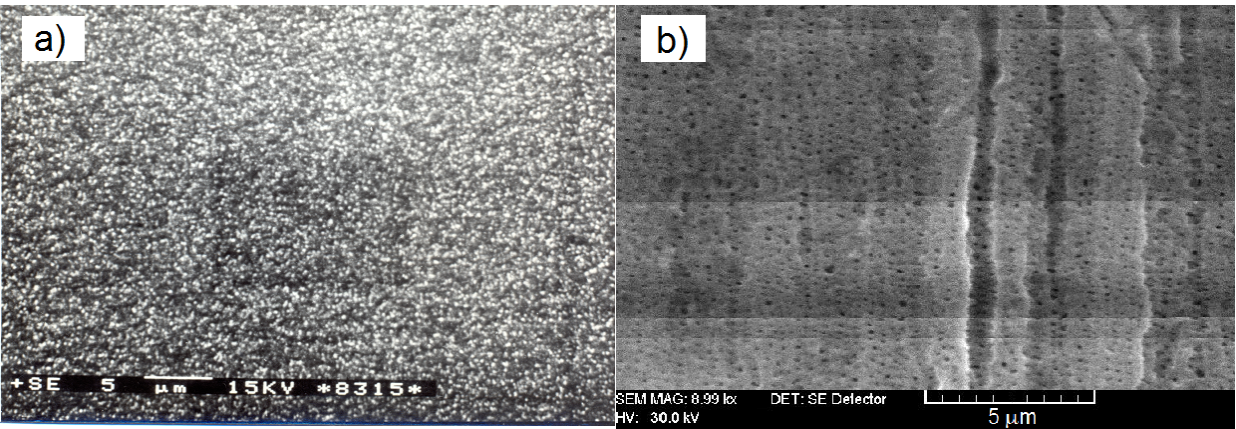
SEM of Te films grown: a) on Pyrex glass at a rate of 10 nm/s and b) on nanostructured Al_2_O_3_ substrates at a rate of 30 nm/s. Scale bar is 5 µm.

Two methods were used to nanostructure Te-based films: growth of Te nanocrystals on Pyrex glass substrates or deposition of amorphous Te films onto nanostructured (porous) Al_2_O_3_ substrates. In both cases, the polycrystalline Te (purity 99.999%) was evaporated under 10^−4^ Pa vacuum conditions. The evaporation was carried out using VUP-5 equipment (SUMI, Ukraine) from a tantalum boat, keeping the same distance (20 cm) between the evaporation boat and substrate, without any cooling or heating of the latter. To grow films with a nanocrystalline structure, a growth rate of about 10 nm/s was used whereas, for amorphous thin films, the growth rate was increased to 30 nm/s. The deposition rate was increased by raising the temperature of the evaporator. The calibration was performed via measuring the final thickness of the grown film versus the time of deposition at given temperature of the evaporator. Under these conditions, the amorphous Te films were grown on either continuous (Pyrex glass) or porous (Al_2_O_3_) nanostructured substrates. Then, rectangular 70 mm^2^ samples were cut from nanocrystalline and nanostructured amorphous Te films, which were either prepared for further morphological and structural analysis or for fabrication of gas-sensitive devices. After preparation, the thickness and shape of the films were studied using a SIS SCAN Control C (PhotoniTech Pte Ltd., Singapore) atomic force microscope. The surface morphology of the films was investigated using either a TESLA BS 340 or a VEGA TESCAN TS 5130 MM (TESCAN, Czech Republic) scanning electron microscope (SEM). To investigate the structural features of the grown films, X-ray analysis was performed using a DRONE–YM1 (Burevestnik, Russia) diffractometer with Fe Kα radiation. The rotational velocity of the scintillation counter was set to be either 2 or 4°/min. For the electrical and gas-sensing characterization, the samples were supplied with symmetrical gold or platinum electrodes, identified in our preliminary works as ohmics [25–26], which form electrically transparent contacts with Te. The gold or copper wires were then attached to the electrodes with a silver paste. Figure 1A shows the surface morphology of a Te film grown on a Pyrex glass substrate at a rate of 10 nm/s. As shown, the film contains a dense nanocrystalline layer with randomly oriented crystals with sizes ranging from 50–100 nm. Figure 1B shows the surface morphology of an amorphous Te film grown on preliminary nanostructured Al_2_O_3_. Pure amorphous films grown on Pyrex glass substrates were uniform and did not exhibit a striking morphological structure; therefore, their SEM image was omitted.

At the same time, the Te films grown on the Al_2_O_3_ substrate (Figure 1B) exhibited a nanostructured morphology, corresponding to the substrate template, which consists of ≈100 nm diameter dotted holes, separated 400 nm from one another. No Te crystallites were observed in this case.

**Figure 2:**
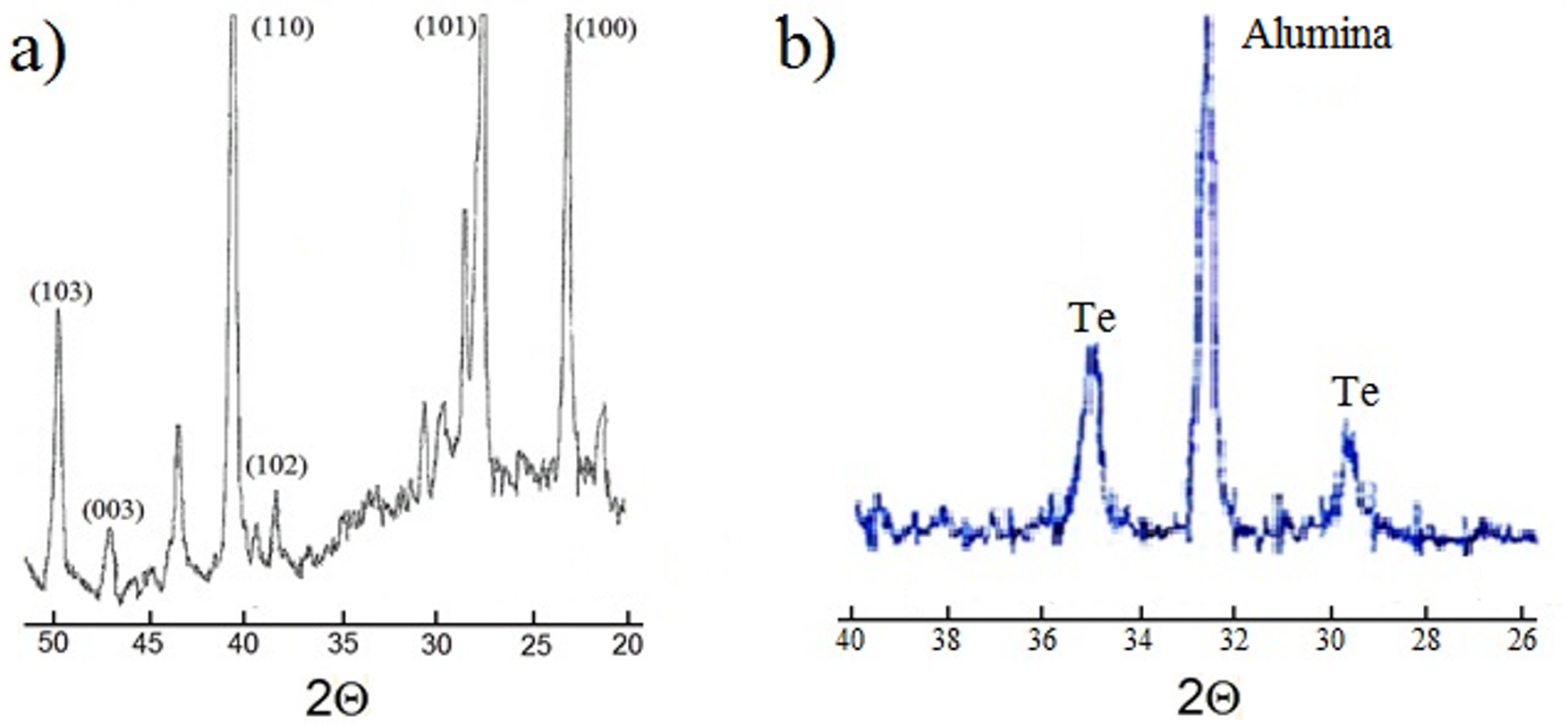
XRD diffraction patterns of Te films grown on Pyrex glass (A) or nanostructured Al_2_O_3_ (B) substrates.

The structural phase state of the grown films was adequately confirmed by X-ray diffraction (XRD). Figure 2 shows the XRD patterns of Te films grown on either Pyrex glass (Figure 2A) or nanostructured Al_2_O_3_ substrates (Figure 2B).

According to Figure 2A, the XRD pattern of films grown on Pyrex glass substrates reveals a highly crystalline structure with a predominant Te hexagonal phase. The positions of the most intense peaks matched the reference values: from right to left, the first peak occurs due to the reflection from the (100) crystal plane, the second peak is observed due to the reflection from the (101) crystal plane and the third peak appears due to the reflection from the (110) crystal plane. The nearly equal intensities of these peaks as well as the appearance of other refraction peaks indicates the absence of a predominant growth orientation of the nanocrystals. As a counter example, the XRD pattern of a Te film grown on Al_2_O_3_ substrates with a higher deposition rate ≈30 nm/s (Figure 2B, adapted from [27]) only shows two weak Te peaks (ASTM, 4-554). These type of films are considered amorphous. It should also be mentioned that the XRD pattern of samples grown on Pyrex glass substrates at the same rate of 30 nm/s (data not shown) does not show features of crystalline Te.

### Gas-sensing characterization, methods and response kinetics

For the gas-sensing characterization of fabricated films, NO_2_ was chosen since it is one of the most active toxic gases known to interact with Te [1].

NO_2_ vapor, with a concentration of either 0.5 or 1.0 ppm, was obtained by using the experimental set up described in our previous paper [28]. Gaseous NO_2_ media was obtained using a calibrated permeation tube (Vici Metronics, USA), which was introduced into the experimental setup. Ambient air was used as both the carrier and the reference gas. Nanostructured Te-based gas-sensing devices were put into a 10 mL test cell and the NO_2_ diluted in ambient air was injected at a 100 mL/min flow rate parallel to the film surface. To perform both the heating and the annealing of the samples, the test cell was mounted inside a furnace. A platinum temperature detector (PT-100, Cliptec Kabeltechnik, Germany) was placed close to the film and was used to assist with the temperature control.

The data was processed using a PC equipped with a data acquisition board (National Instruments Inc., USA). Characteristic transient current response curves were collected at a constant applied voltage (5 V), using different NO_2_ concentrations at different temperatures. The switching between the mixture of NO_2_ vapor and reference gases was computer-controlled. The time delay between measurements was 2 s, which was, simultaneously, much smaller than the sensor response time and much higher than the assessed dielectric relaxation time value. In order to transform the resistance signal into a voltage signal, the sample was connected in series to a load resistance using a dc voltage supplier. In all measurements, the load resistance was chosen to be approximately one order of magnitude lower than the sample resistance.

**Figure 3:**
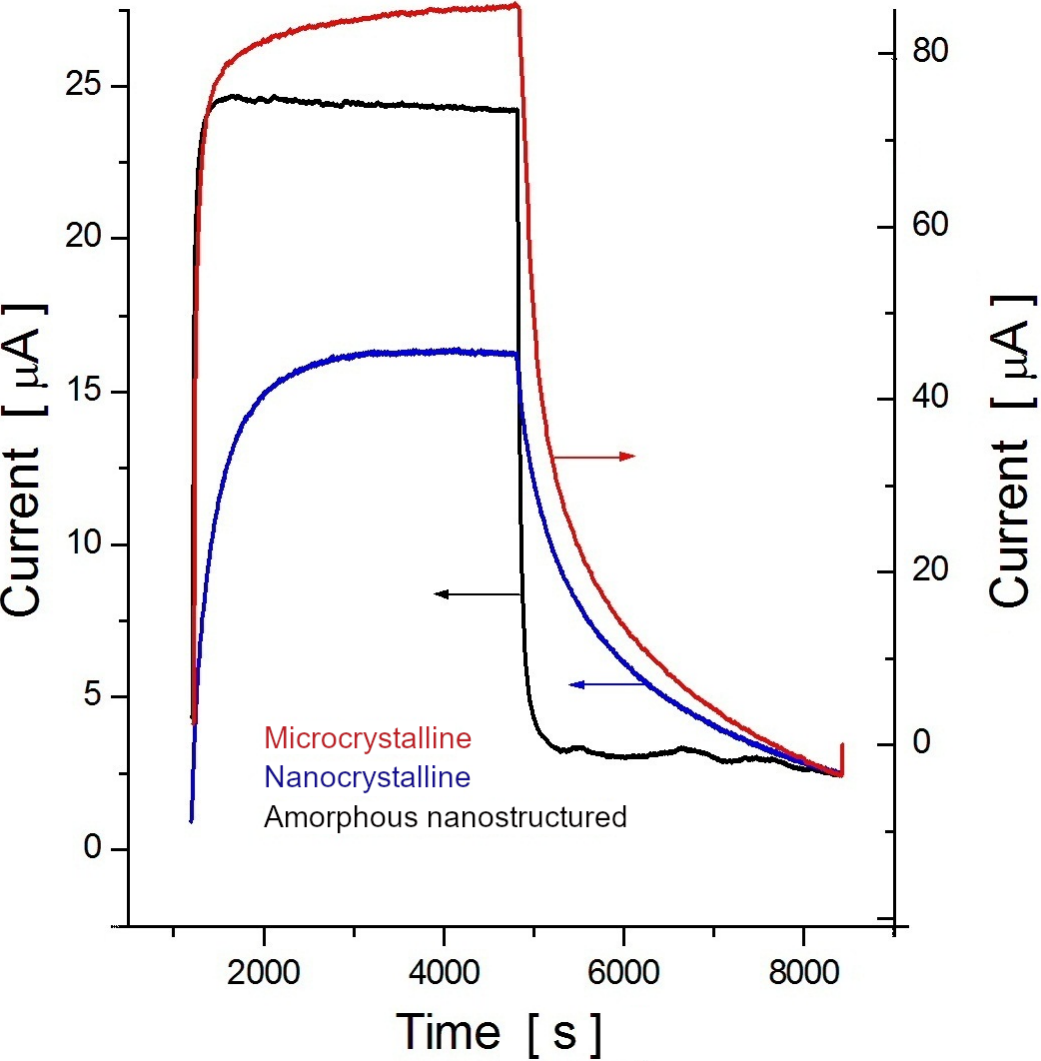
Normalized dynamic response of a microcrystalline (red), nanocrystalline (blue) and amorphous (black) nanostructured Te-based gas-sensitive device to a rectangular pulse of 1.0 ppm NO_2_ at room temperature (22 °C).

Figure 3 shows the dynamic response of both nanocrystalline (blue) and amorphous (black) nanostructured Te-based gas-sensitive devices to a concentration pulse of 1.0 ppm NO_2_ at room temperature (22 °C). As a comparison, and under the same conditions, the response of a microcrystalline film grown on a Pyrex glass substrate at a deposition rate of 1 nm/s was also added. The morphological and structural features of microcrystalline films were previously described in detail [5–6]. It can be observed in Figure 3 that both the response current and the time to reach the saturation decrease with the reduction of the crystallite structural block dimension, i.e., from microcrystalline (red) to nanocrystalline (blue) states. On the other hand, the behavior of the nanostructured amorphous films (black curve) is significantly different from the crystalline ones. It is worth noting that these parameters also depend on the film thickness, temperature and gas concentration.

### The effect of temperature and gas concentration on nanocrystalline films

**Figure 4:**
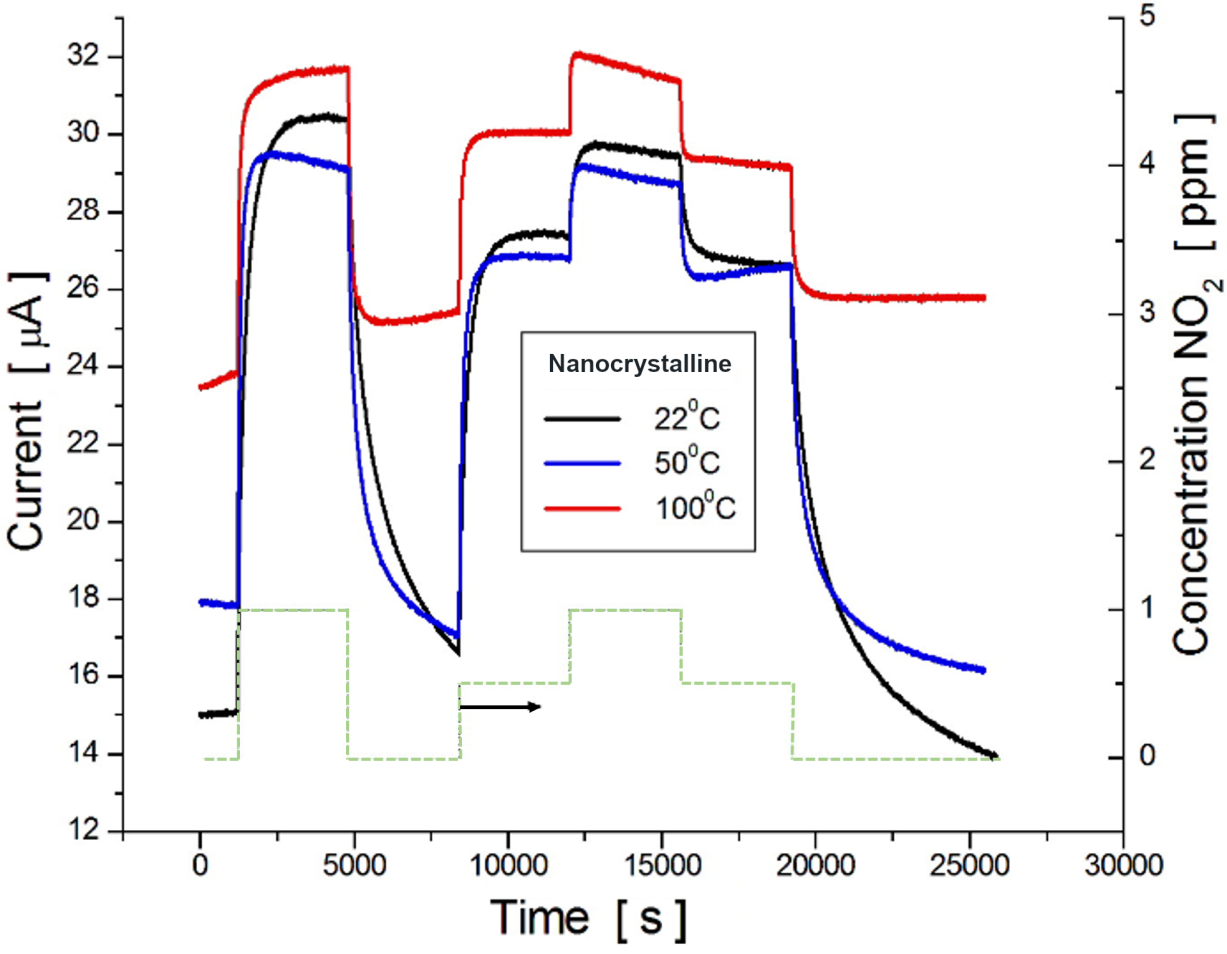
Transient characteristics of gas-induced current in nanocrystalline Te films, at different temperatures, due to the exposure to various NO_2_ concentrations, according to the profile shown by the green dashed line at the bottom.

Figure 4 shows the current flow through a ≈100 nm thick nanocrystalline film submitted to repeated switching on–off cycles of the NO_2_ gas mixture at a constant bias voltage and 22 °C operating temperature. Square pulses of NO_2_ vapor at concentrations of 0 ppm, 0.5 ppm, and 1.0 ppm were applied. The dashed green line shows the switching profile. It is seen that the current follows the same pattern but the baseline strongly increases with temperature, as depicted in Table 1. Table 1 also shows the film sensitivity, which is calculated from the response kinetics to 1.0 ppm NO_2_, as a relative percent increase in current (in %/ppm) according to Equation 1:

[1] $$S=100(I_{\text{g}}-I_{\text{a}})/C\cdot I_{\text{g}}$$

where *I*_a_ and *I*_g_ are the currents flowing through the specimen in air and in the presence of NO_2_, respectively, and *C* is the gas concentration.

**Table 1:** Gas-sensing parameters of nanostructured Te films. *t*_rv_ is the recovery time and *t*_rs_ is the response time upon exposure to 1.0 ppm of NO_2_.

Films	*T* [°C]	Baseline [µA]	Sensitivity [%/ppm]	*t*_rs_ [s]	*t*_rv_ [s]

nanocrystalline	22	15	50	160	600
	50	18	40	70	200
	100	24	25	50	50
amorphous nanostructured	22	11	65	15	30

Figure 4 shows that, independent of the operating temperature, the recovery time (*t*_rv_) is longer than the response time (*t*_rs_). These parameters, listed in Table 1, were estimated to be the time to reach and to lose 50% of the maximum value of *I*_g_.

According to Table 1, both *t*_rs_ and *t*_rv_ decrease with the temperature increase; however, the sensitivity of the films diminishes. In the following subsection, interesting results will be explored in terms of solving this sensitivity issue by nanostructuring amorphous Te-based films.

### Amorphous nanostructured films

**Figure 5:**
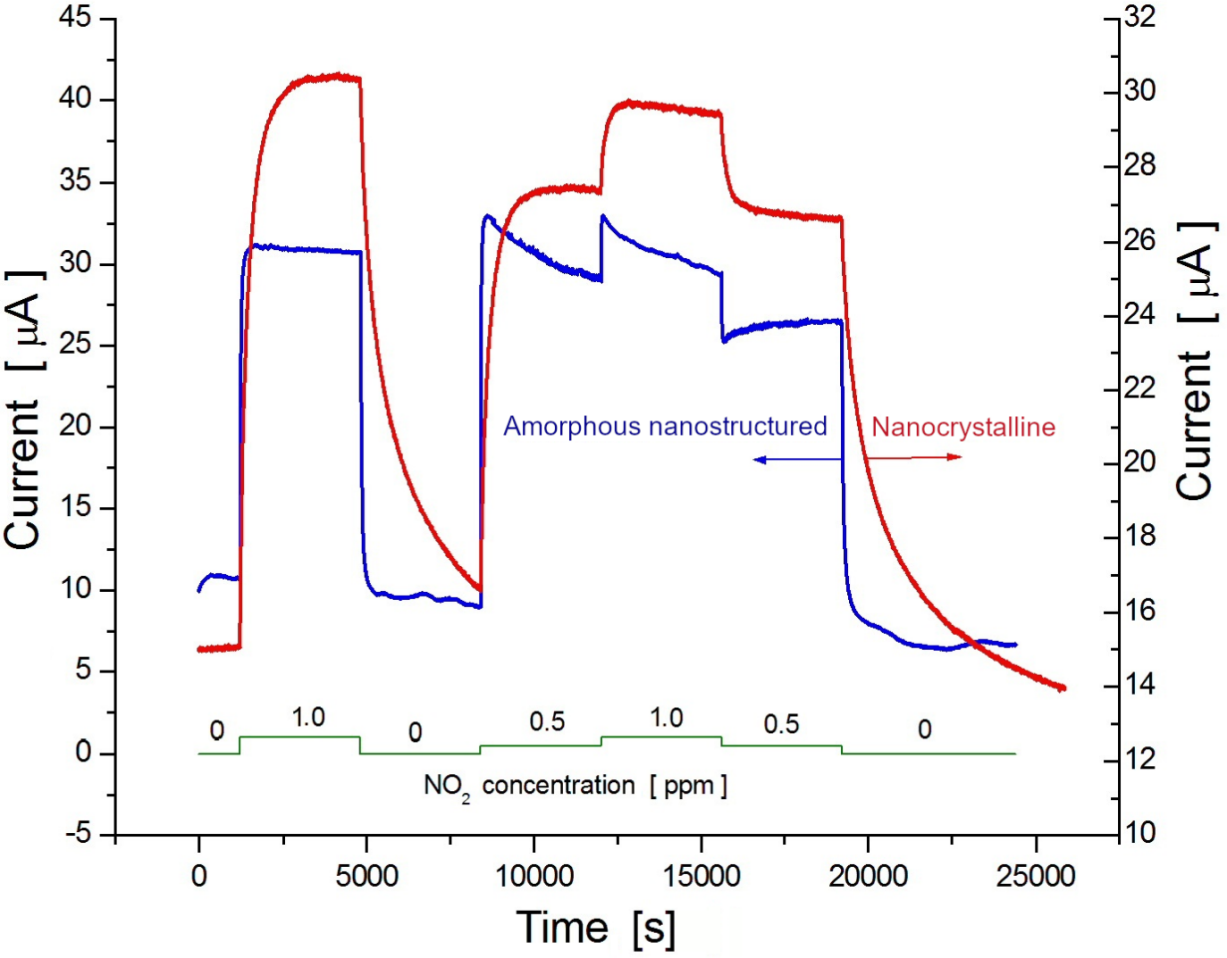
Transient characteristics of gas-induced current in amorphous nanostructured Te films (blue curve) at room temperature (22 °C) due to exposure to various concentrations of NO_2_, according to the profile shown by the green continuous line at the bottom. The transient characteristic profile for nanocrystalline film (red curve) is given for comparison.

**Figure 6:**
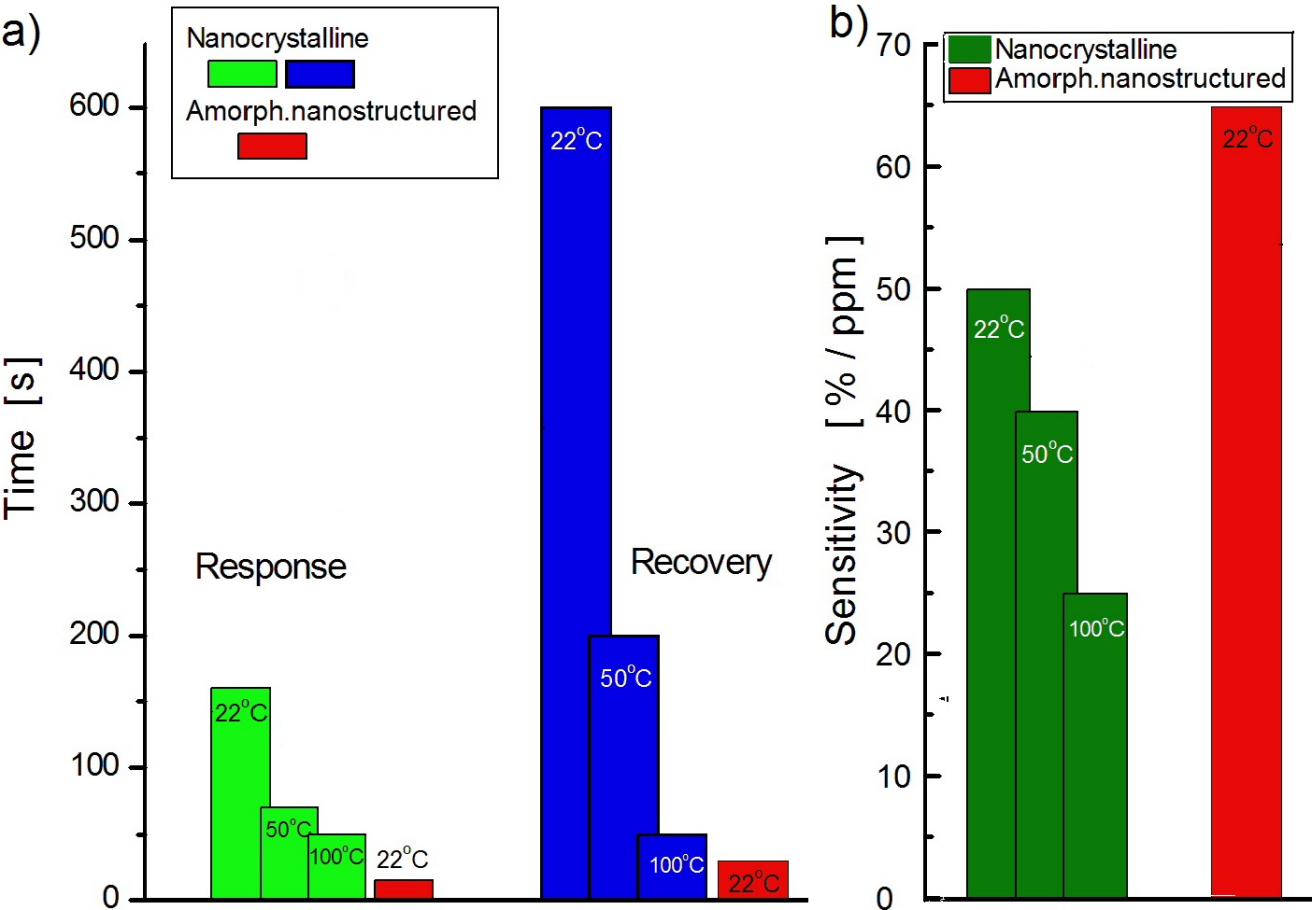
Comparison of the gas-sensing properties of nanocrystalline and amorphous nanostructured Te-based films to NO_2_ at different temperatures: A) response and recovery times; B) sensitivity.

Figure 5 illustrates the transient characteristics of the gas-induced current in both nanocrystalline Te films, grown on Pyrex glass substrate, and amorphous Te films, grown on a nanostructured Al_2_O_3_ substrate. The NO_2_ exposure profile curve is shown by the green continuous line at the bottom. The operating temperature was maintained at 22 °C. There is no noticeable baseline drift in either the nanocrystalline or in the amorphous nanostructured films; however, a dramatic change in the response kinetics can be clearly observed. These changes consist of a decrease in both the response and recovery times for amorphous Te films grown on preliminary nanostructured Al_2_O_3_ substrates. As shown in Figure 6A, in such films, the response time is approximately only 15 s whereas the recovery time is about twice as long (see red bars). Interestingly, the reduction in both the response and recovery times is accompanied by an impressive increase in gas sensitivity reaching 65%/ppm, which is the highest among the values obtained for the nanostructured films studied. The diagram presented in Figure 6B illustrates this result.

Another peculiarity of the transient characteristics of amorphous nanostructured films is a spontaneous reduction (or increase) in the gas-induced current in terms of step change concentration of the target gas.

## Discussion

This paper presents a study regarding two different types of nanostructured Te films physically built either in the form of nanocrystals, grown onto flat substrates, or vitreous Te, deposited onto nanostructured (porous) dielectric templates. It was expected that the physical properties, including the adsorptive ones, of these films would differ from each other and also from those observed in the microcrystalline Te-based films. In fact, this assumption, previously mentioned in our review paper [29], was confirmed by our results presented in Figure 3, which shows the normalized response kinetics to the target gas NO_2_ when in contact with microcrystalline, nanocrystalline or amorphous nanostructured Te-based films. Since that review was dedicated to exploring only the fabrication and investigation of gas-sensing properties of nanocrystalline Te films, it was only superficially mentioned that the amorphous films could improve the response kinetic parameters in detriment of sensitivity. One way to enhance both the response time and sensitivity to NO_2_ seems to be the mechanical nanostructuring of amorphous Te films, which is investigated and discussed in this paper. So far, nanocrystalline Te films have been grown using either the thermal vacuum evaporation of pure Te [17–20] or its sputtering under a pure argon atmosphere [24] onto glass, Al_2_O_3_ or sapphire substrates. It was shown that the film morphology as well as the gas sensitivity is controlled by several factors, such as the nature and temperature of the substrate, the type and concentration of the target gas molecules, film thickness, post-preparation thermal treatment and operating temperature. It is more or less generally accepted that the films grown on glass substrates at room temperature exhibit a maximum sensitivity to gases such as NO_2_, H_2_S or ammonia. This is the main reason why this method was used in this work to manufacture and examine Te films. According to the SEM image shown in Figure 1A, nanocrystalline Te films grown on glass substrates present a uniform and dense distribution of randomly oriented nanocrystalline grains with an approximate average size of 100 nm. Assuming the neutrality of the Au/Te contacts, the electrical conductivity of the film is mainly controlled by the bulk, surface and grain boundary resistances. On the other hand, due to the peculiarities of chalcogens and chalcogenide materials [1,30], a region enriched in holes is formed at the surface and at grain boundary and intragrain regions. Therefore, when the films are exposed to NO_2_, the surface and grain boundaries are the most affected by the gas reaction. Although the gas sensing occurs due to the variation in hole density at the enriched region (surface and grain boundary), in the presence of gaseous media, the bulk is responsible for the observed increase in the baseline current when the temperature increases (Figure 4). Elevated temperatures result in the decrease of the gas (NO_2_) sensitivity, as shown in Figure 6B. At the same time, the increase in the operating temperature values affects the gas response kinetics (Figure 4), essentially decreasing both the response and recovery times (Figure 6A). The decrease in both the response and recovery times with an increase in temperature can be partially explained by Maxwell’s dielectric relaxation time (τ_r_). As τ_r_ = εε_0_ρ (ρ is the bulk resistivity, ε and ε_0_ are the permittivity and the electric constant, respectively), it is clear that τ_r_ decreases since there is a reduction in the resistivity when the temperature increase and the system reaches steady state in less time. Another suitable explanation for the reduction in the response and recovery times with the increase in temperature might be the shift in the adsorption–desorption equilibrium, pointed out in Langmuir's theory [31].

The nanostructuring of Te-based films by growing amorphous Te on nanoporous Al_2_O_3_ substrates (Figure 1B) allows for a reduction in the response and recovery times and a simultaneous increase in the sensitivity to NO_2_. As show in Figure 6A and Table 1, both the response and recovery times for these films are, respectively, more than 10 and 20 times shorter in comparison to the nanocrystalline film kinetics. In addition, the sensitivity to 1 ppm of NO_2_ increases by 15%/ppm. It is worth noting that such remarkable improvement in gas sensing parameters is achieved without heating, since the working temperature is kept at 22 °C (room temperature). The reason for such behavior seems to be due to the increase in the chemical activity of chalcogenides at the surface when disorder (amorphization) increases [32–33] and to the increase in the active area caused by substrate porosity. Another interesting feature observed in the experiments was the spontaneous reduction (or sometimes increase) in the gas-induced current upon step-change concentration of the target gas. This can be explained based on the concentration-induced phenomenon that induces sensitivity damping in ultrathin films [34]. It is safe to assume that, given the high rate at which the films are grown (30 nm/s), their thickness remains low (<40 nm) since the film is deposited on the walls of the porous surface. On the one hand, these ultrathin films show a considerably short response time, but on the other hand, the increase in gas concentration damps their sensitivity due to the formation of a catalytic gate at the surface.

## Conclusion

The nanostructuring of Te-based films by growing vitreous Te on a pre-nanostructured (porous) dielectric template significantly improves their gas sensing capabilities. At room temperature (22 °C), the response and recovery times decrease by approximately 10 and 20 times, respectively, in comparison with nanocrystalline Te films. In addition, there is an increase in the gas sensitivity by 15%/ppm. These achievements can be attributed to two main factors: the increase in chemical activity of chalcogenides at the surface due to increase in disordering (amorphization) and an increase in the active surface area due to increased substrate porosity.

## References

[1] Tsiulyanu D, Reithmaier J, Paunovic P, Kulisch W (2011). Tellurium Thin Films in Sensor Technology. Nanotechnological Basis for Advanced Sensors. NATO Science for Peace and Security Series B: Physics and Biophysics.

[2] He Z, Yang Y, Liu J-W, Yu S-H (2017). Chem Soc Rev.

[3] Ba L A, Döring M, Jamier V, Jacob C (2010). Org Biomol Chem.

[4] Szaro L (1986). Thin Solid Films.

[5] Tsiulyanu D, Stratan I, Tsiulyanu A, Liess H-D, Eisele I (2007). Sens Actuators, B.

[6] Tsiulyanu D, Marian S, Miron V, Liess H-D (2001). Sens Actuators, B.

[7] Siciliano T, Filippo E, Genga A, Micocci G, Siciliano M, Tepore A (2009). Sens Actuators, B.

[8] Bianchetti M F, Heredia E, Oviedo C, Walsöe de Reca N E (2005). J Argent Chem Soc.

[9] Sen S, Muthe K P, Joshi N, Gadkari S C, Gupta S K, Jagannath, Roy M, Deshpande S K, Yakhmi J V (2004). Sens Actuators, B.

[10] Sen S, Bhandarkar V, Muthe K P, Roy M, Deshpande S K, Aiyer R C, Gupta S K, Yakhmi J V, Sahni V C (2006). Sens Actuators, B.

[11] Tsiulyanu D, Marian S, Liess H-D (2002). Sens Actuators, B.

[12] Wang S, Wen H, Guan W, Zhang L, Ma D, Huang S, Wang J (2010). Thin Solid Films.

[13] Liang F, Qian H (2009). Mater Chem Phys.

[14] Ma J, Liu X, Wu L, Zheng W (2008). Cryst Res Technol.

[15] Rheem Y, Chang C H, Hangarter C M, Park D Y, Lee K H, Jeong Y S, Myung N V (2010). Electrochim Acta.

[16] Thiebaud L, Legeai S, Stein N (2016). Electrochim Acta.

[17] Sen S, Bhatta U M, Kumar V, Muthe K P, Bhattacharya S, Gupta S K, Yakhmi J V (2008). Cryst Growth Des.

[18] Tsiulyanu D, Marian S, Liess H-D, Eisele I (2004). Sens Actuators, B.

[19] Bhandarkar V, Sen S, Muthe K P, Kaur M, Kumar M S, Deshpande S K, Gupta S K, Yakhmi J V, Sahni V C (2006). Mater Sci Eng, B.

[20] Tsiulyanu D, Marian T, Tiuleanu A, Liess H-D, Eisele I (2009). Thin Solid Films.

[21] Her Y C, Huang S L (2013). Nanotechnology.

[22] Kumar V, Sen S, Sharma M, Muthe K P, Jagannath, Gaur N K, Gupta S K (2009). J Nanosci Nanotechnol.

[23] Wang Z, Wang L, Huang J, Wang H, Pan L, Wei X (2010). J Mater Chem.

[24] Siciliano T, Di Giulio M, Tepore M, Filippo E, Micocci G, Tepore A (2008). Sens Actuators, B.

[25] Ciobanu M, Tsiulyanu D, Kantser V, Andronic S (2015). Electric conductivity of tellurium-based chalcogenides thin films with Au and Ag contacts. ICTEI-2015: International Conference on Telecommunications, Electronics and Informatics. Proceedings.

[26] Ciobanu M, Tsiulyanu D (2018). Chalcogenide Lett.

[27] Tsiulyanu D, Mocreac O (2018). Impedance characterization of Te based gas sensitive films. Proceedings 6th International Conference on Telecomunications, Electronics and Informatics.

[28] Tsiulyanu D, Ciobanu M (2019). Glass Phys Chem.

[29] Tsiulyanu D, Moraru A, Petkov P, Tsiulyanu D, Kulisch W (2015). Nanocrystalline Tellurium Films: Fabrication and Gas Sensing Properties. Nanoscience Advances in CBRN Agents Detection, Information and Energy Security.

[30] Mott N F, Davis E A (1979). Electronic processes in non-crystalline materials.

[31] Wolkenstein T (1987). Electronic Processes on Semiconductor Surfaces During Chemosorption.

[32] Ciobanu M (2018). Contacts and surface phenomena in quaternary glassy chalcogenides based on S and Te.

[33] Mamontova T N, Kochemirovskii A S, Pivovarova L V (1988). Phys Status Solidi A.

[34] Tsiulyanu D, Mocreac O (2013). Sens Actuators, B.

